# Catalytic Performance of Toluene Combustion over Pt Nanoparticles Supported on Pore-Modified Macro-Meso-Microporous Zeolite Foam

**DOI:** 10.3390/nano10010030

**Published:** 2019-12-20

**Authors:** Sibei Zou, Mingyuan Zhang, Shengpeng Mo, Hairong Cheng, Mingli Fu, Peirong Chen, Limin Chen, Wei Shi, Daiqi Ye

**Affiliations:** 1 School of Environment and Energy, South China University of Technology, Guangzhou 510006, China; es2017zousibei@mail.scut.edu.cn (S.Z.); 201710106086@mail.scut.edu.cn (M.Z.); moshengpeng14@mails.ucas.ac.cn (S.M.); mschenghairong@mail.scut.edu.cn (H.C.); mlfu@scut.edu.cn (M.F.); chenpr@scut.edu.cn (P.C.); liminchen@scut.edu.cn (L.C.); 2 National Engineering Laboratory for VOCs Pollution Control Technology and Equipment, Guangzhou Higher Education Mega Centre, Guangzhou 510006, China; 3 Guangdong Provincial Key Laboratory of Atmospheric Environment and Pollution Control (SCUT), Guangzhou Higher Education Mega Centre, Guangzhou 510006, China

**Keywords:** hierarchically porous zeolite foam, Pt nanoparticles, diffusion, acid etching, catalytic combustion of toluene

## Abstract

Herein, to investigate the pore effect on toluene catalytic oxidation activity, novel supports for Pt nanoparticles—ZSM-5 foam (ZF) fabricated using polyurethane foam (PUF) templates and pore-modified ZSM-5 foam (ZF-D) treated by acid etching, comparing with conventional ZSM-5 and pore-modified ZSM-5 (ZSM-5-D), were successfully synthesized. Pt nanoparticles were loaded on series ZSM-5 supports by the impregnation method. The Pt loaded on ZF-D (Pt/ZF-D) showed the highest activity of toluene catalytic combustion (i.e., T_90_ = 158 °C), with extraordinary stability and an anti-coking ability. Based on various catalysts characterizations, the unique macropores of ZF facilitated the process of acid etching as compared to conventional ZSM-5. The mesopores volume of ZF-D significantly increased due to acid etching, which enlarged toluene adsorption capacity and led to a better Pt distribution since some Pt nanoparticles were immobilized into some mesopores. Specifically, the microporous distribution was centered in the range of 0.7–0.8 nm close to the molecular diameter of toluene (ca. 0.67 nm), which was key to the increasing toluene diffusion rate due to pore levitation effect of catalysts and accessibility of metal. Furthermore, the reducibility of Pt nanoparticles was improved on Pt/ZF-D, which enhanced the activity of toluene catalytic oxidation.

## 1. Introduction

Volatile organic compounds (VOCs), as one of the most important precursors of air pollution for causing many environmental problems, are generated by the vehicle exhaust emission, industrial processes emission, and volatile chemical products [1,2,3,4,5]. Over the past several decades, developing techniques such as adsorption [6,7], catalytic combustion [8], thermal incineration [9], biological processes, plasma catalytic oxidation [10], and photocatalytic degradation, etc. have attracted great attention to remove VOCs. Among them, catalytic combustion is a well-known technology of high catalytic performance, economic feasibility, high selectivity, low operation cost, and non-organic by-products for its approximately completely catalytic oxidation of VOCs into CO_2_ and H_2_O at low temperatures [11,12,13]. According to the previous studies, it is reported that lower reaction temperature is more economical for the catalytic oxidation of VOCs [14] so that noble-metal catalysts with high activity, high selectivity, and low activation energies have attracted much attention [15]. Li’s group also reported that supported noble-metal-based catalysts show much better low-temperature catalytic efficiency than non-noble-metal-based catalysts [3]. Specifically, Pt-based catalysts are widely applied for total catalytic oxidation of VOCs due to their extraordinary low-temperature activities [16].

Nowadays, enhancing the catalytic efficiency of noble metal catalysts and the stability of noble metal nanoparticles are still urgent problems that need to be overcome because of the high cost of noble metal and their easy aggregation [17,18]. In general, supports with large surface area play significant roles in metal distribution and the adsorption of reactants during the catalytic combustion [16]. It has been evidenced that zeolite-supported Platinum Group Metal (PGM) nanoparticles have become one of the most efficient catalysts in recent years [19,20,21]. ZSM-5, as one of the most important high-silica microporous zeolites, is commonly utilized as supports in the catalytic oxidation of VOCs due to its unique intricate channels, excellent hydrothermal stability, and high adsorption capacity [22]. Nevertheless, in the conventional ZSM-5, its relatively small micropores and narrow channels (ca. 0.5 nm) hinder the intra-crystalline diffusion of large molecules such as toluene (ca. 0.67 nm) [23] in the catalytic reaction, which might lead to coke formation and deactivation of catalysts [5,24].

As the previous studies reported, for the levitation effect, Yashonath [25] reported that, when the pore size is close to the molecular diameter of adsorbates, the diffusion energy barrier will be greatly reduced, which plays a significant role in the diffusion of adsorbates. It is significant to control suitable pore sizes of zeolites, which can be beneficial for the adsorption and diffusion of VOC molecules and the accessibility of the active sites during the catalytic combustion. Numerous recent studies have been devoted to introducing mesoporous structures into the zeolite by many different strategies, such as dealumination and desilication, to uniform the distribution of noble metal [18] and maximize the synergy of mass transfer between mesopores and micropores. Previously, a monolithic ZSM-5 foam (ZF) has been successfully synthesized by the utilization of polyurethane foams (PUFs) as templates, which could be an ideal support for active noble metal catalysts because of its highly ramified networks of interconnecting macropores for easier internal molecular diffusion [26]. Moreover, we expected that the zeolite foam could also be favorable for the acid dealumination of zeolite with unique large macropores.

Herein, we report a supported PGM catalyst of Pt loading on the pore-modified ZSM-5 foam (ZF-D) with hierarchical porosity (macro-meso-micropore) using the incipient wetness impregnation technique, exhibiting an excellent catalytic activity of toluene combustion with long catalysts lifetimes. The hierarchical porous ZF-D was successfully synthesized by the acid etching dealumination owing to the unique macropores of ZSM-5 zeolite foam, in comparison to the conventional ZSM-5. The key to its superior catalytic performance is the appearance of suitable micropores and mesopores that promotes the diffusion and adsorption capacity of toluene, and the defects (mesopores) that are favorable for distribution of Pt nanoparticles. These results will be in a more detailed description by various characterization analyses in this paper.

## 2. Materials and Methods 

### 2.1. Materials

ZF samples with a Si/Al ratio of 53 (the Si/Al ratio was determined from ICP-OES) were synthesized according to the literature [26]. In the typical synthesis of ZF: tetraethyl orthosilicate (TEOS, Aladdin, Shanghai, China, GC, >99%, 17 g) and tetra propylammonium hydroxide (TPAOH, Aladdin, 2 M in H_2_O, 5 mL) were mixed with H_2_O (54 mL) by stirring. Suitable NaAlO_2_ (Aladdin, technical) was slowly added to the resultant gel and then stirred at the room temperature for 4 h. The molar composition of the gel was 80 SiO_2_; 6.2 Al_2_O_3_; 10 TPAOH; 3000 H_2_O. The gel finally turned clear and was transferred to an autoclave containing the PUF template. The PUF was frequently squeezed and fully drained with tweezers during the introduction of the gel. In our case, the size of the PUF was 2.5 mm × 2.5 mm × 7 mm. The hydrothermal synthesis was conducted at 140 °C for 48 h. After the hydrothermal synthesis, the ZSM-5 was finally crystallized on the PUF. The synthesized monolith was washed with deionized water and dried at room temperature, then calcined at 550 °C for 5 h under the airflow to remove the organic templates. The monolith with calcination was labeled as ZF.

The ZF-D was dealuminated from ZF by HNO_3_ acid etching. Add a certain amount of ZF monolith with HNO_3_ (Guangzhou Chemical Reagent Factory, Guangzhou, China, AR, 65–68%, 50 mL) at a solid-to-liquid ratio about 1:50 into a Teflon-lined autoclave and heated at 80 °C for 24 h. The product was collected after water washing until the PH ≈ 7. The obtained product was dried at 100 °C for 12 h, which was still a monolith, labeled as ZF-D.

Conventional ZSM-5 with Si/Al ratio of 59 (the Si/Al ratio was determined from ICP-OES) was synthesized by a typical preparation method. As the same method as ZF, except for PUF templates, the products were calcined at 550 °C for 5 h under the airflow, labeled as ZSM-5. For comparison, the pore-modified ZSM-5 was prepared the same way as the defected ZF, labeled as ZSM-5-D.

Pt loaded zeolite catalysts (Pt/ZF-D, Pt/ZF, Pt/ZSM-5-D, Pt/ZSM-5) were all prepared by simple incipient wetness impregnation synthesis in the same way. ZF and ZF-D supports were ground into powder before the impregnation. Take Pt/ZF-D as an example. An aqueous solution containing a certain amount of Pt(NO_3_)_2_ (10%, Xiya Reagent, Shandong, China) was added into 1 g ZF-D support. The mixture was then stirred until completely dried and calcined at 550 °C for 5 h. After reducing with 5% H_2_/Ar flow (50 mL/min) at 500 °C for 2 h, Pt/ZF-D was finally obtained. All of the catalysts were in the form of powder. The Pt loadings in the samples determined from ICP-OES measurements are presented in Table S1.

### 2.2. Catalytic Characterization

The crystal lattice structure of the samples was determined by the powder X-ray diffraction (XRD) patterns with a Bruker D8 ADVANCE diffractometer (Bruker, Billerica, MA, USA, Cu Kα, λ = 1.54056, 40 kV, 40 mA, scanning step = 0.02°). The data was collected at a scan rate of 10° min^−1^ within the range of scattering angle 2θ of 5° to 90°. The specific surface area and pore size distributions of all the samples were determined by the N_2_ adsorption–desorption on an automatic surface analyzer (Micromeritics ASAP 2020, Micromeritics Instrument Corporation, Norcross, GA, USA). Before measurement, each sample was outgassed at 120 °C for 3 h. Morphologies of samples were determined by field-emission scanning electron microscopy (FESEM, JEOL JSM-6700F, JEOL Ltd., Tokyo, Japan) using an electron microscope. Si/Al ratios of zeolites and Pt contents in catalysts were determined by inductively coupled plasma with a PerkinElmer plasma 8000 optical emission spectrometer (ICP-OES, PerkinElmer, Inc., Waltham, MA, USA). X-ray photoelectron spectroscopy (XPS) analysis was performed using a Thermo ESCALAB 250Xi electron spectrometer (Thermo Fisher Scientific Inc., Waltham, MA, USA) with Al Kα (hν = 1486.8 eV) as the excitation source. Binding energies of all elements were referenced to the C 1s line at 284.8 eV from carbon impurities. The measurement of toluene diffusion over the prepared zeolites was conducted by a computer-controlled intelligent gravimetric analyzer (IGA-003, Hiden Analytical Ltd., Warrington, UK). The toluene vapor was supplied to the zeolite samples at 30 °C and the toluene adsorption isotherms were continuously recorded until 250 °C. The coke amount of spent catalysts after 50 h long-time tests was analyzed by Thermogravimetric analysis (TGA Q50, TA Instruments, New Castle, DE, USA). Toluene-Temperature programmed desorption (TPD) experiments were carried out in a continuous N_2_ flow (100 mL/min). The sample (ca.100 mg) was placed in a fixed-bed quartz tubular and was exposed to the flow of toluene/N_2_ for 12 h before the TPD experiments. The toluene/N_2_ vapor with a total flow rate of 100 mL/min containing 1000 ppm toluene was generated by bubbling N_2_ through a bottle containing pure toluene. Then, it was swept under N_2_ flow for 1 h to remove physically adsorbed molecules. The TPD was performed from 25 to 500 °C with a rate of 5 °C min^−1^ and maintaining at the final temperature for about 30 min. The effluent gases were continuously monitored by on-line Hiden HPR-20 QIC TMS Mass Spectrometry (Hiden Analytical Ltd., Warrington, UK). Transmission electron microscope (TEM) images were taken in a JEM-2100HR (JEOL Ltd., Tokyo, Japan). The mean size of the controllable Pt nanoparticles was determined from TEM images of the as-prepared Pt particles. The equation was as follows:

(1) $$d\approx \frac{\sum_{i}n_{i}d_{i}}{\sum_{i}n_{i}}$$

where $n_{i}$ is the nanoparticle number and $d_{i}$ is the diameter of the nanoparticle. Moreover, it is assumed that the Pt nanoparticles showed a spherical shape, so the dispersion of the Pt nanoparticles could be estimated based on the following equation [8]:

(2) $$D_{\mathrm{Pt}}=\frac{600M_{\mathrm{Pt}}}{\rho {\mathrm{da}}_{\mathrm{Pt}}N_{A}},$$

where D_Pt_ is the dispersion of the Pt nanoparticles (%), 600 is a value with no unit, M_Pt_ denotes the molar weight of Pt (195.08 g mol^−1^), ρ is the density of Pt (21.45 g cm^−3^), d (nm) is the average particle diameter observed from the TEM images, a_Pt_ represents the surface area of Pt atom (8.06 × 10^−20^ m^2^ atom^−1^), and N_A_ is the Avogadro constant (6.02 × 10^23^ mol^−1^).

### 2.3. Catalytic Evaluation

The catalytic activity of the catalysts was evaluated under atmospheric pressure under steady-state conditions in a fixed-bed quartz tubular micro-reactor (6 mm i.d., 500 mm length). In addition, 100 mg catalysts and 400 mg of quartz sand were mixed well and filled quartz wool to the quartz reactor at both ends of the catalyst bed to minimize the effect of hot spots. The reactant gases (1000 ppm toluene, 20 vol.% O_2_ + balance N_2_) passed through the reactor at a rate of 100 mL min^−1^, and the corresponding space velocity (SV) was 60,000 mL g^−1^h^−1^. The concentration of reactant and product gases was analyzed using an online gas chromatograph (GC-2014C, Shimadzu, Kyoto, Japan) equipped with a flame ionization detector (FID). Before the outlet products were measured by the gas chromatograph at a given temperature 8 times, the toluene oxidation reaction was stabilized for 15 min. The catalytic activity of the samples was evaluated using the temperature (T_50_, T_90_) required to achieve toluene conversions of 50% and 90%, respectively. The range of catalytic activity testing temperatures was 100 °C to 220 °C. The catalytic activities over the catalysts were calculated according to the following equation [8,27]:

(3) $$X_{toluene}=\frac{C_{toluene,in}{-C}_{toluene,out}}{C_{toluene,in}}\times 100\%$$

where X_toluene_, C_toluene, in_ (ppm), and C_toluene, out_ (ppm) are the toluene conversion, toluene concentration in the inlet and outlet gas, respectively.

The toluene catalytic combustion rates (rate_toluene_) and TOF, defined as the number of toluene molecules converted per second on each exposed Pt (Pt^0^) species in Pt-based catalysts, can be calculated according to the following equation [28]:

(4) $$rate_{toluene}=\frac{X_{Toluene}\left[\%\right]\times F_{Toluene}\left[L_{Toluene}\cdot min^{-1}\right]}{m_{catal}\left[g\right]\times 60\left[s\cdot min^{-1}\right]\times 22.4\left[L\cdot mol^{-1}\right]}\left[mol_{Toluene}\cdot g_{catal}\cdot s^{-1}\right]$$

(5) $$TOF=\frac{rate_{Toluene}\left[mol_{Toluene}\cdot g_{catal}\cdot S^{-1}\right]\times M_{Pt}\left[g\cdot mol^{-1}\right]}{D_{Pt}\left[\%\right]}\left[s^{-1}\right]$$

where X_Toluene_ is the conversion of toluene (Equation (3)), F_Toluene_ is the volumetric flow rate of toluene, m_catal_ is the mass of catalysts, and D_pt_ is the dispersion of Pt nanoparticles based on the TEM result (Equation (2)).

## 3. Results and Discussion

### 3.1. Catalytic Performance

#### 3.1.1. Catalytic Combustion of Toluene over Pt/ZF-D, Pt/ZF, Pt/ZSM-5-D, and Pt/ZSM-5

**Figure 1:**
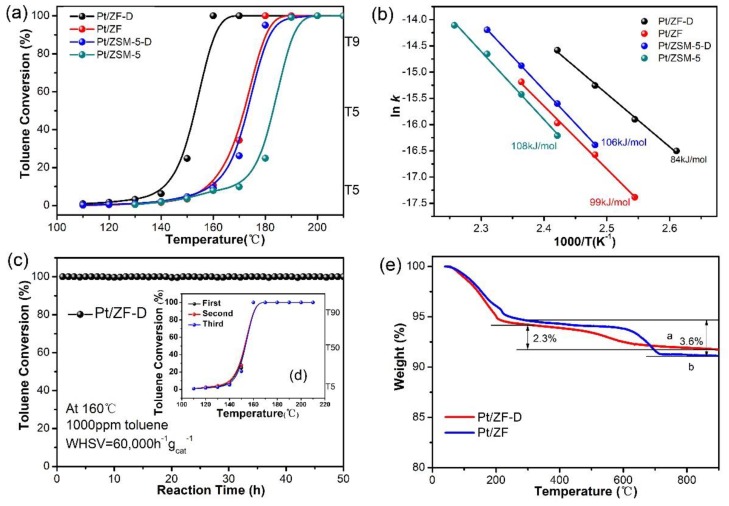
Catalytic performance, stability, repeated tests, and activation energies of all the catalysts: (**a**) catalytic combustion performance and (**b**) Arrhenius plots of Pt/ZF−D, Pt/ZF, Pt/ZSM−5, and Pt/ZSM−5−D under the conditions of toluene concentration at 1000 ppm and Weight Hourly Space Velocity (WHSV) at 60,000 mL/(g h); (**c**) catalytic activity stability tests during 50 h reaction time over Pt/ZF−D; (**d**) conversion of toluene over Pt/ZF−D with three repeated catalytic runs; (**e**) coke formation condition after stability tests: TGA curves of Pt/ZF−D and Pt/ZF after the catalytic combustion of toluene for 50 h under the conditions of toluene concentration at 1000 ppm, WHSV at 60,000 mL/(g h), and reaction temperature at 160 °C of Pt/ZF−D and 180 °C of Pt/ZF, respectively.

Figure 1a shows catalytic activities of toluene over the Pt/ZF-D, Pt/ZF, Pt/ZSM-5-D, and Pt/ZSM-5 (ca. 0.5%Pt loading, ICP analysis, Table S1) catalysts. All samples performed high activities, which could completely decompose toluene below 190 °C. To compare catalytic activities, T_5_, T_50_, and T_90_ of toluene conversions were estimated from Figure 1a, as listed in Table S2. T_90_ of toluene was achieved at 158 °C over Pt/ZF-D which was about 20, 21, and 30 °C lower than that of Pt/ZF, Pt/ZSM-5-D and Pt/ZSM-5 (178, 179, and 188 °C), respectively. The T_5_ and T_50_ of Pt/ZF-D were approximately 135 and 152 °C, respectively, which were also lower than that of Pt/ZF, Pt/ZSM-5-D, and Pt/ZSM-5. The activity of the Pt/ZF-D catalyst was the highest one.

Arrhenius plots of these samples are exhibited in Figure 1b. The activation energies of all the catalysts were calculated from Arrhenius plots and the formula, as listed in Table S2. In addition, the relevant toluene reaction rate to calculate the activation energies were listed in Table S3. The E_a_ values follow the sequence: Pt/ZF-D (84 kJ) < Pt/ZF (99 kJ) < Pt/ZSM-5-D (106 kJ) < Pt/ZSM-5 (108 kJ). The catalyst with a lower E_a_ value would present easier complete oxidation and higher catalytic activity for toluene. Combining the catalytic performance of toluene combustion with the results of the kinetic evaluation, it can be confirmed that the Pt/ZF-D sample exhibited the best catalytic performance for toluene oxidation among all catalysts. The activation energies of catalysts were calculated under 10% conversion rates of toluene. Since the low-temperature activities of these catalysts were similar, there was no big change in the activation energies of catalysts, especially among Pt/ZF, Pt/ZSM-5-D, and Pt/ZSM-5.

For practical application, both the catalytic lifetime and stability are significant parameters for measuring the application potential of a catalyst. Figure 1c shows the long-term trajectory of the catalytic activities on the reaction time of 50 h in the toluene combustion at 160 °C over the best-performing catalyst Pt/ZF-D. In this reaction, nearly no CO was detected over all of the catalysts during the reaction, in which CO_2_ and H_2_O were the only products and the selectivity of CO_2_ in the products was approximately 100% (Figure S1 and more details in Supplementary Materials). It is found that all catalysts exhibited approximately 100% conversion of toluene. This result indicates that all the catalysts have long catalytic life towards toluene combustion. Figure 1d shows three repeated catalytic runs of toluene complete oxidation over Pt/ZF-D. It is observed that the three-run curves overlapped very well, indicating that the stability of Pt/ZF-D is so excellent even after three times repeated catalytic reaction.

TGA curves of the Pt/ZF-D and Pt/ZF after 50 h reaction time are shown in Figure 1e. The weight loss before 300 °C is mainly assigned to water desorption, while the weight loss at relatively high temperatures (300–900 °C) could be assigned to coke formation during the reaction [16,24]. Comparatively, the weight loss at 300–900 °C of Pt/ZF-D (2.3%) was much less than that of Pt/ZF (3.6%), indicating that Pt/ZF had much stronger coke formation than Pt/ZF-D. In addition, the surface and morphology between the fresh Pt/ZF-D and the spent Pt/ZF-D after 50h reaction time showed no differences (Figure S2), which could further confirm that Pt/ZF-D showed an excellent anti-coking ability. This phenomenon should be attributed to the fact that Pt/ZF-D is favorable for mass transfer in the catalytic combustion of toluene.

#### 3.1.2. Catalytic Combustion of Toluene over xPt/ZF-D (Where x = 0.1%, 0.5%, 1%, 2%Pt Loadings)

**Figure 2:**
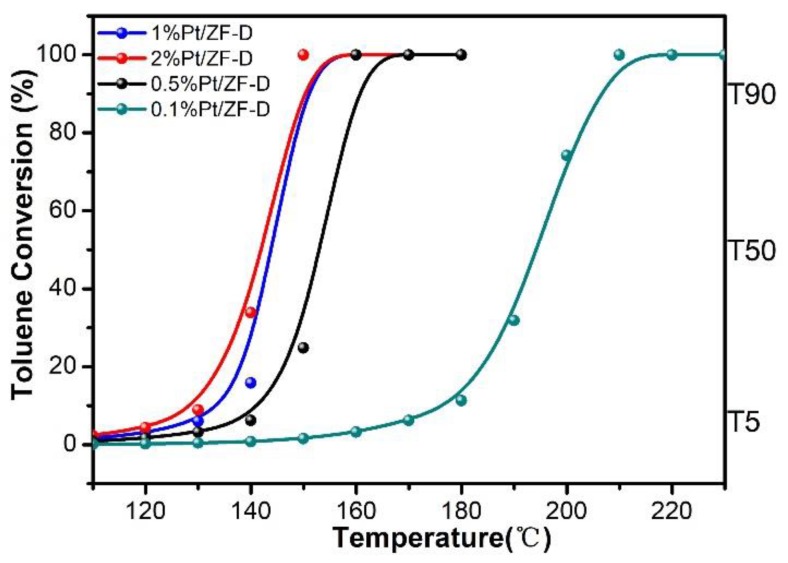
xPt/ZF-D (where x = 0.1%, 0.5%, 1%, and 2%Pt loadings) catalytic combustion of toluene under the conditions of toluene concentration at 1000 ppm and WHSV at 60,000 mL/(g h).

To evaluate the effect of hierarchical porous structure on ZF-D, the catalytic combustion of toluene over xPt/ZF-D with different Pt loadings were conducted. As shown in Figure 2, it could be observed that all samples performed a high activity, which could decompose toluene completely below 210 °C. To compare their catalytic activities, T_5_, T_50_, and T_90_ of toluene conversions were estimated from Figure 2 as listed in Table 1. It can be proved that Pt loadings play a significant role in the enhancement of activity. With Pt loading from 0.1% to 1%, the activities of catalysts were increased as the Pt loading increased. For example, T_90_ of toluene conversion was only 148 °C over 1%Pt/ZF-D, which was about 10 and 57 °C lower than that of 0.5%Pt/ZF-D and 0.1%Pt/ZF-D (158 and 205 °C, respectively). T_5_ and T_50_ of 1%Pt/ZF-D were 126 and 144 °C, respectively, which was also lower than that of 0.5%Pt/ZF-D and 0.1%Pt/ZF-D. However, with Pt loadings from 1% to 2%, the activities of catalysts were only slightly improved. As shown in the supplementary information (Figure S3 and Table S4), it might be attributed to the larger Pt particles and lower Pt dispersion in 2%Pt/ZF-D.

It should be noted that xPt/ZF-D (where x = 1%, 0.5%, 0.1%Pt loadings) catalysts had excellent activities of toluene combustion. Compared with similar previous studies [5,16,24,29,30] (as listed in Table 1), we find that the T_90_ of both 1% and 0.5%Pt/ZF-D showed more extraordinary activities than other studies. Considering both the high price and high catalytic activity of the noble metal, the 0.5% Pt loading might be more mainstream. Interestingly, 0.5%Pt/ZF-D was lower than many previously studied zeolite-supported catalysts by nearly 20 °C or more over the same Pt loading level. However, this phenomenon does not indicate whether the zeolite foam or the concentrated HNO_3_ acid etching plays a major role. To explain such a great improvement of toluene combustion activity, characterization experiments for the same loadings of Pt (0.5%) on different supports (ZF-D, ZF, ZSM-5, and ZSM-5-D) were then conducted.

### 3.2. Catalysts Characterization

#### 3.2.1. Structural and Morphology Analysis

**Figure 3:**
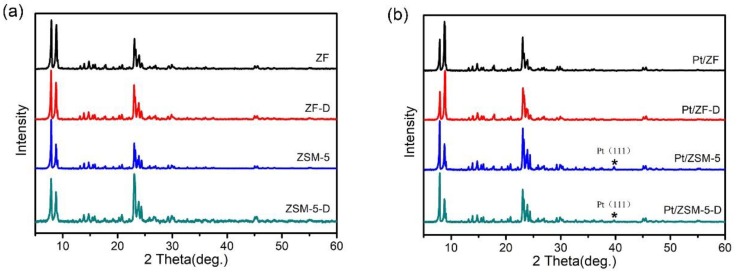
The structural analyses of zeolites and catalysts: XRD patterns of (**a**) ZSM-5, ZSM-5-D, ZF, and ZF-D samples; (**b**) Pt/ZSM-5, Pt/ZSM-5-D, Pt/ZF, and Pt/ZF-D samples. All the samples exhibit the typical MFI zeolite structure.

The as-obtained samples were investigated by XRD to confirm their structural properties, as shown in Figure 3. According to 2θ from 7° to 10° and from 22° to 25°, both the zeolite supports and the Pt-based catalysts exhibit well-resolved characteristic peaks associated with a typical MFI zeolite structure [31]. It is indicated that the acid etching did not destruct the MFI zeolite structure of ZF-D and ZSM-D. Based on the ICP analysis (Table S2), the Si/Al ratio between ZF and ZF-D increases to 58.59%, while the one between ZSM-5 and ZSM-5-D is 25.42%, which means that the dealumination degree of ZF-D is higher than ZSM-5-D. As shown in Figure 3b, the characteristic peaks of metallic platinum (2θ at 39.8° and 46.2°) cannot be observed in the XRD patterns between Pt/ZF-D and Pt/ZF probably because of the low loading of Pt and high dispersion of Pt species. However, the weak diffraction peak of Pt (111) plane at 39.8° over Pt/ZSM-5-D and Pt/ZSM-5 can be observed probably because of the formation of Pt nanoparticles’ aggregation. It is proposed that Pt dispersion may be different on the ZF and ZSM-5 series supports, which could be resulted from the dealumination degree and the effect of hierarchically porosity. The XRD characterization in Figure S4a showed no differences in typical MFI zeolite structures and no characteristic peaks of metallic platinum between the fresh Pt/ZF-D and the spent Pt/ZF-D, indicating no changes of the structural properties and no obvious Pt nanoparticles agglomeration after the catalytic reaction.

**Figure 4:**
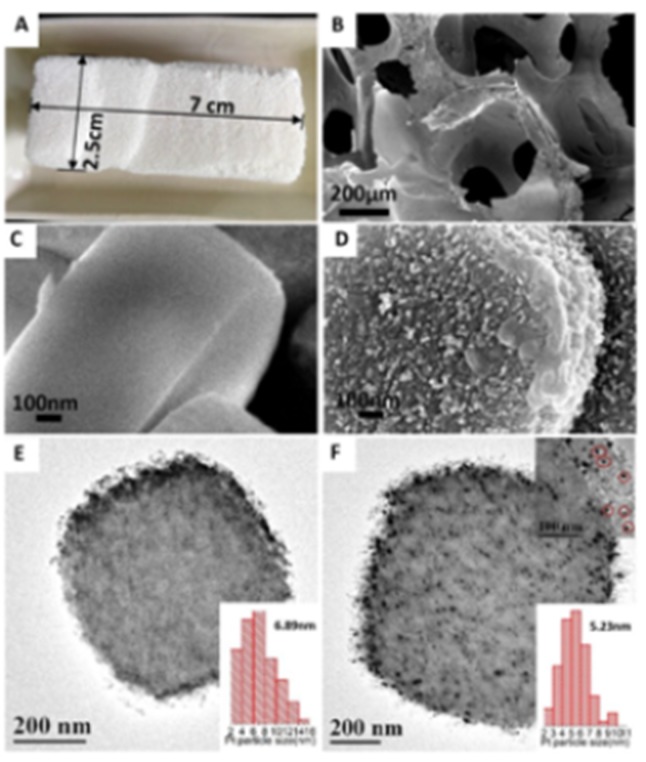
The morphology analyses of the samples: (**A**) the photographs of ZF. SEM images of (**B**) ZF macropores; (**C**) ZF crystal surface; and (**D**) ZF-D crystal surface. The macropores of the ZSM-5 foam are stacked by a pile of ZSM-5 crystals. The surface of the acid-etching ZF-D crystal turn obviously rough compared to ZF. TEM images of (**E**) Pt/ZF, (**F**) Pt/ZF-D (red circles highlight Pt particles immobilized into defects).

To explore the differences between ZF and the conventional ZSM-5, as well as the influence of the nitric acid etching, SEM and TEM characterizations were carried out, as shown in Figure 4, Figures S5 and S6. As shown in Figure 4A, after synthesis and calcination at 550 °C, the ZF did not shrink and perfectly maintained the cuboid-shape and size of the PUF template, indicating the formation of fully-grown monolithic foam [32]. The ZF sample exhibited macropores ranging from 100–200 μm that can be observed in Figure 4B. Interestingly, the macro-pores of the ZF are composed of the accumulation of numbers of orthorhombic shaped ZSM-5 crystals. The crystal size of ZF is ca.1.5 μm according to the characterization of SEM. After treating ZF by HNO_3_, the surface of the zeolite crystals turned rough from smooth as shown in Figure 4C,D, indicating that HNO_3_ acid-etched resulted in the dealumination of ZF, etching the surface of crystals and creating abundant defects on the surface successfully. A similar result could be also observed between ZSM-5 and ZSM-5-D (Figure S5). Due to the unique loose macropores structure in ZF, the ZF-D surface was uniformly etched by nitric acid to defects of about 5–20 nm (Figure 4F), and most of the Pt nanoparticles are immobilized into these defects of ZF-D, which the red circles highlight. Pt nanoparticles with the mean Pt nanoparticle size of 5.23 nm were uniformly dispersed throughout the Pt/ZF-D (Figure 4F). However, the Pt particles were agglomerated in both Pt/ZSM-5-D and Pt/ZSM-5 as shown in Figure S6, with very low Pt dispersion (Table 2). According to the previous studies [33,34,35], the weak metal–support interactions facilitate the migration of metallic particles. Therefore, comparatively, the ZF crystals have stronger metal–support interactions with smaller size and higher dispersion of Pt nanoparticles that are more stable. It is confirmed that high Pt dispersion plays a significant role in the total toluene combustion. According to the Pt dispersion, we calculated the TOF values of catalysts at 140 °C as listed in Table S2. However, the TOF values with respect to the exposed Pt atom were very different but didn’t show a positive correlation with Pt particle size. This result indicates that the Pt particle size is not the only active site to control the reaction. In addition, the adsorption and diffusion in the zeolite pores might have a positive effect on the best catalytic performance of Pt/ZF-D which will be further confirmed.

#### 3.2.2. Surface Compositions Analysis

**Figure 5:**
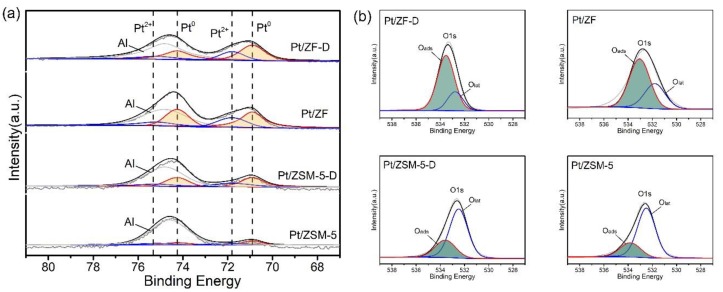
XPS spectra of (**a**) Pt 4f and (**b**) O 1s peaks of Pt/ZF-D, Pt/ZF, Pt/ZSM-5-D and Pt/ZSM-5 catalysts.

Furthermore, to learn the surface compositions of samples, the XPS characterization was conducted, as shown in Figure 5, Table 2 and Table S5. Since Al 2p peaks strongly overlapped with Pt 4f peaks in the range of 68–80 eV, it was necessary to separate Al 2p peaks from these spectra. In this case, Al 2p peaks were used at 74.8 eV in Figure 5a. The Pt 4f_7/2_ spectra were deconvoluted into two peaks at 70.9 eV as well as 71.8 eV while the Pt 4f_5/2_ spectra were deconvoluted into two peaks at 74.25 eV together with 75.15 eV. The peaks at 70.9 eV and 74.25 eV were assigned to the Pt^0^ species and the peaks at 71.8 eV and 75.15 eV were assigned to the Pt^2+^ species, which were very consistent with those in pieces of literature [24,30,36]. After deconvolution, the Pt^0^ content of catalysts followed in a sequence of Pt/ZF-D (67.3%) > Pt/ZF (65.1%) > Pt/ZSM-5-D (62.2%) > Pt/ZSM-5 (46.6%) (in Table 2 and Table S4). This result indicated that the presence of mesopores was more beneficial to the formation of Pt^0^ species at ZF samples than ZSM-5 samples. Although it could not be the main reason for the high activities of the catalysts, the high proportion of Pt^0^ in Pt/ZF-D is beneficial to the total toluene combustion.

In addition, the O1s peaks of the catalysts are shown in Figure 5b. As a previous study reported [5], O 1s peak with higher binding energy could be assigned to loosely bounded surface oxygen species (O_ads_), and the peak with lower binding energy could be assigned to surface lattice oxygen species (O_lat_). The O_ads_/O_lat_ atomic ratios of catalysts positively decreased in the sequence of Pt/ZF-D (3.38) > Pt/ZF (1.38) > Pt/ZSM-5-D (0.38) > Pt/ZSM-5 (0.33), which were consistent with their Pt^0^ content. Among all the catalysts, Pt/ZF-D had the highest O_ads_/O_lat_ atomic ratios, confirming that increases in both Pt dispersion and Pt^0^ proportion are favorable for the existence of the largest amount of reactive oxygen species.

#### 3.2.3. Pore Size Distribution Analysis

**Figure 6:**
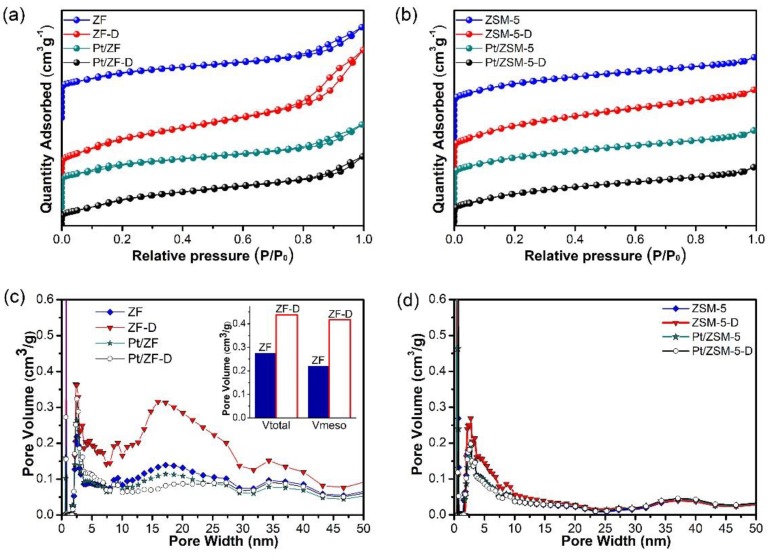
N_2_ physisorption isotherms and pore width distributions of all the samples: (**a**) The typical type IV adsorption isotherms with hysteresis loops (H4 type) suggest the mesopores structures of the ZF zeolite series samples, while (**b**) suggest almost none mesopores appear in ZSM-5 zeolite series samples. The pore width distribution curves of (**c**) ZF zeolite series samples exhibit more mesopores appearing in the pore range of 2–50 nm in ZF-D than in ZF. Thus, the pore volume of ZF-D is significantly enlarged, compared to ZF without acid-etching. (**d**) The pore width distribution curves of ZSM-5 zeolite series samples show only a slight increase in mesopores after the acid etching of ZSM-5.

The N_2_ adsorption–desorption experiment was conducted to explore the texture structure of these samples, as shown in Figure 6a. The ZF zeolite series samples presented typical type IV adsorption isotherms with hysteresis loops (H4 type), while the ZSM-5 zeolite series samples (in Figure 6b) presented typical type I isotherms according to the Brunauer-Deming-Deming-Teller (BDDT) classification [37]. After being acid-etched by HNO_3_, enlarged hysteresis loops (H4 type) at *P/P*_0_ over ZF-D could be obviously observed, which was assigned to capillary condensation owing to the existence of mesopores that were successfully created by the HNO_3_ etching [38,39,40].

In pore size distribution curves (Figure 6c), both ZF&ZF-D (ZFs as short below) and ZSM-5&ZSM-5-D (ZSM-5s as short below) had the highest intensity peaks in the micropores range of below 1 nm, which corresponded to the MFI zeolite structure [41]. Microporous distribution of ZFs with a relatively intensive peak was centered in 0.75 nm, which was close to the diameter of toluene (ca. 0.67 nm), as shown in Figure S7. As the previous study reported, for the levitation effect, Yashonath [25] reported that, when the pore size is close to the molecular diameter of adsorbates, the diffusion energy barrier will be greatly reduced, which plays a significant role in the diffusion of adsorbates. It is indicated that micropores in ZFs are advantageous to the adsorption capacity towards toluene, which can be also beneficial to toluene complete oxidation. In addition, it will be further confirmed in the next part of the paper.

In the pore width range of 2–50 nm (Figure 6d), ZF-D showed more mesopores structures than ZF. It might be further confirmed that defects (extra mesopores) of ZF-D were successfully formed by dealumination, which could be clearly seen in both SEM and TEM images. Correspondingly, the Brunauer-Emmett-Teller (BET) surface area (Table 3) becomes larger after nitric acid etching, which could be matched with this result. The presence of extra mesopores enlarged the total pore volume of Pt/ZF-D catalysts, which were favorable for the diffusion of reactants and products, and the dispersion of Pt nanoparticles [5]. Interestingly, ZFs exhibited a border peak at the range of 2–50 nm than that of ZSM-5s, and the former average mesoporous width was also larger than the latter one. Due to the unique macropores structures in ZF, the process of acid etching significantly created more mesopores in ZF-D than ZSM-5-D as compared with the one in ordinary ZSM-5 without the macropores structures (in Figure 6c,d). In addition, according to the mesoporous volume in Table 3, the mesoporous structure of ZF-D was nearly twice as much as ZF. As a result, the mesoporous structure of Pt/ZF-D catalyst is more favorable for the distribution of Pt nanoparticles, which can be confirmed by the result of the TEM characterization.

Results of N_2_ adsorption–desorption indicate that typical hierarchical porous structures and the enlarged pore volume of Pt/ZF-D can be beneficial for the diffusion and adsorption of the relatively large molecules such as toluene. This result can be further confirmed by IGA and Toluene-TPD analysis. Compared to the pure supports, the surface areas and pore volumes of catalysts slightly decrease due to the block of zeolites channel, which confirms that Pt particles have been perfectly immobilized on the surface and mesopores of these supports, increasing the Pt dispersion. As shown in Figure S4b,c, both the fresh and spent 0.5%Pt/ZF-D catalysts presented the typical type IV adsorption isotherms with hysteresis loops (H4 type) and nearly the same pore distribution. It is further confirmed that there was no significant change of the catalysts’ physicochemical properties after toluene combustion, combined with the XRD characterization.

#### 3.2.4. The Mechanism of Toluene Diffusion in the Hierarchical Porous Catalysts

**Figure 7:**
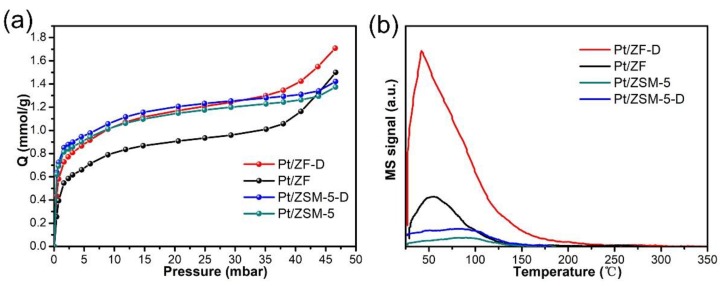
(**a**) the adsorption isotherms of toluene over Pt/ZF-D, Pt/ZF, Pt/ZSM-5-Dand Pt/ZSM-5 catalysts by IGA at 30 °C; (**b**) toluene adsorption–desorption profiles of Pt/ZF-D, Pt/ZF, Pt/ZSM-5-D, and Pt/ZSM-5.

To explore whether the specific micropores or mesopores in Pt/ZF-D are beneficial for the diffusion of toluene is necessary. In addition, combining with the XPS results, the slight increase of Pt^0^ content could not comprehensively explain the obvious increase of toluene catalytic performance between Pt/ZF-D, Pt/ZF, and Pt/ZSM-5-D. The probable reason is the considerable enhancement of active site accessibility [16]. Toluene adsorption was conducted by IGA analysis on the different catalysts at 30 °C. As shown in Figure 7a, the toluene adsorption isotherms of Pt/ZF-D and Pt/ZF exhibited Type-IV and increased along with the pressure, demonstrating the presence of mesoporous structure, while both Pt/ZSM-5-D and Pt/ZSM-5 exhibited typical Langmuir Type-I adsorption [42]. Pt/ZF-D possessed the largest adsorption capacity of toluene among all the catalysts. For the Pt loaded zeolite, the diffusion coefficients (*D*) of toluene were calculated following Fick’s law (in the Supplementary Materials), as summarized in Table 4. In general, the adsorption diffusion coefficients (*D_ads_*) of Pt/ZF-D and Pt/ZF were 10 times more than that of Pt/ZSM-5-D and Pt/ZSM-5 at all the pressures. *D_ads_* of Pt/ZF-D were 2.08 and 2.59 times that of Pt/ZF at 2 and 1 mbar, while *D_ads_* of Pt/ZSM-5-D were 1.17 and 1.18 times that of Pt/ZSM-5, respectively. These above results further confirmed that the typical hierarchical porous structure of Pt/ZF-D significantly improved the diffusion rate of toluene owing to the toluene condensation in the specific micropores of 0.7–0.8 nm. In addition, the defecting process enhanced the mesoporous structure with the promoted diffusion and the metal accessibility in Pt/ZF-D, which could be an inherent reason for its improving catalytic combustion of toluene. This result is also consistent with the results of BET and XPS.

As the previous study reported, toluene could be used to probe the adsorption and diffusion of reactants [43]. We then conducted the toluene adsorption–desorption (Toluene-TPD) experiments to further explore the ability of catalysts for the adsorption and diffusion of toluene. As shown in Figure 7b, all the samples showed one toluene desorption peak at between 50 °C and 220 °C, respectively. The toluene desorption temperature of Pt/ZF-D was the highest among the four catalysts, which might mean tighter binding between the toluene molecule and the catalyst. Obviously, the toluene desorption peak intensity of Pt/ZF-D was also the highest compared with the four catalysts, which can be assigned to its strongest adsorption of toluene and the largest amount of toluene storage capacity. Therefore, the tight binding between the toluene and Pt/ZF-D, the strongest adsorption of toluene, as well as the largest amount of toluene storage capacity could be another significant reason for the improving catalytic activity. This phenomenon could mainly result from the hierarchical porosity and enlarged pore volume of ZF-D supported catalysts.

## 4. Conclusions

In summary, ZSM-5 foam (ZF) and pore-modified ZF (ZF-D) supported Pt catalysts were studied in this work. Hierarchically macro-meso-microporous ZF-D was successfully synthesized by a nitric acid etching strategy, and Pt nanoparticles were loaded onto ZF and ZF-D respectively using the wetness impregnation method. It was confirmed that the surface of the ZF-D crystal was fabricated with defects via the nitric acid-etching, in which macro-porous structures of the ZF played a significant role. Consequently, an obvious increase in mesopores at the range of 2–50 nm of ZF-D was shown in the N_2_ adsorption–desorption characterization, compared with ZF. The micropore size of the ZF-D was centered in the range of 0.7–0.8 nm close to the molecular diameter of toluene (0.67 nm). Combining the results of IGA and Toluene-TPD analyses, the typical hierarchical porous structures of Pt/ZF-D significantly improved the diffusion rate of toluene. The micropores in ZF-D were advantageous to the toluene adsorption capacity, and the defecting process enhanced the mesoporous structure and promoted the diffusion and the metal accessibility in Pt/ZF-D. The toluene adsorption diffusion coefficients (D_ads_) of Pt/ZF-D were 2.08 and 2.59 times that of Pt/ZF at 2 and 1 mbar, which was corresponding to the high catalytic activity of Pt/ZF-D. Moreover, TEM and XPS analyses involved the fact that the hierarchical porous structures could improve the Pt nanoparticle dispersion and Pt^0^ proportion effectively. Compared with Pt/ZF, Pt/ZSM-5-D, and Pt/ZSM-5 catalysts, Pt/ZF-D exhibited the highest catalytic performance of toluene combustion (T_90_ = 158 °C). In addition, Pt/ZF-D also displayed excellent long-term stability (50 h), consecutive catalytic stability, and anti-coking ability, which were three important performances for its industrial application. Considering both the high price and high activity of the noble metal, the 0.5 wt% Pt loading was the optimal Pt loading of this work—with higher activity and lower price. This work provides a strategy to design a hierarchical porous (macro-meso-micropores) structure catalyst using acid etching ZSM-5 foam zeolite. The importance of hierarchical pore modification, which will have a great prospect of catalytic industrial application in the near future on the catalytic performance of VOC combustion over Pt/acid etching zeolite foam, has been proved in this work.

## Figures and Tables

**Table 1:** Catalytic performance over various catalysts for toluene combustion.

Catalysts	Activities (°C) ^a^			Ref.	Pt loadings ^b,c^
	T_5_	T_50_	T_90_		
2%Pt/ZF-D	122	141	148	This work	2.054% ^b^
1%Pt/ZF-D	126	144	148	This work	1.02% ^b^
0.5%Pt/ZF-D	135	152	158	This work	0.52% ^b^
0.1%Pt/ZF-D	166	194	205	This work	0.084% ^b^
Pt/HPMOR	-	175	190	[5]	1% ^c^
Pt-1.9/ZSM-5	138	147	155	[29]	1% ^c^
Pt-1.9/ZSM-5	161	172	175	[29]	0.5% ^c^
Pt@PZN-2	-	-	176	[16]	0.45% ^c^
Pt-R/Beta-H	121	186	195	[24]	-
Pt/HZSM-5–60	165	182	205	[30]	0.5% ^c^

^a^ SV of all catalytic combustion is 60,000 mL/(g h), toluene concentration of all catalytic combustion is 1000 ppm. ^b^ Determined by ICP-OES in this work. ^c^ Determined by the references.

**Table 2:** Chemical properties of the Pt/ZF-D, Pt/ZF, Pt/ZSM-5-D, and Pt/ZSM-5 Catalysts.

Catalysts	T_90_ (°C)	E_a_ (kJ/mol) ^a^	Pt loadings ^b^/%	Oads/Olat ^c^	P(Pt0) ^d^	Pt particle size ^e^	Pt dispersion ^f^
Pt/ZF-D	158	84	0.084	3.38	67.3%	5.23 nm	21.5%
Pt/ZF	178	99	0.58	1.38	65.1%	6.89 nm	16.3%
Pt/ZSM-5-D	179	107	1.02	0.38	62.2%	30 nm	4.5%
Pt/ZSM-5	188	109	2.05	0.33	46.6%	50 nm	2.2%

^a^ E_a_ values were calculated under 10% conversion rates of toluene. ^b^ Pt loadings were determined by ICP-OES. ^c^ O_ads_/O_lat_ was calculated by XPS analysis. ^d^ Proportion of Pt^0^ was calculated by XPS analysis. ^e^ Pt particle size was calculated by TEM. ^f^ Pt dispersion was calculated by Equation (2).

**Table 3:** Physicochemical properties of supports and catalysts.

Samples	*S_BET_*^a^ (m^2^/g)	*S_ext_*^b^ (m^2^/g)	*V_total_*^c^ (cm^3^/g)	*V_micro_*^d^ (cm^3^/g)	*V_meso_*^e^ (cm^3^/g)
ZF	329	303	0.2749	0.059	0.2209
ZF-D	402	364	0.4382	0.021	0.4172
ZSM-5	383	195	0.2551	0.097	0.1581
ZSM-5-D	419	266	0.2938	0.079	0.2148
Pt/ZF	287	230	0.2518	0.033	0.2188
Pt/ZF-D	390	275	0.3089	0.061	0.2469
Pt/ZSM-5	378	186	0.2548	0.099	0.1558
Pt/ZSM-5-D	390	192	0.2595	0.100	0.1595

^a^*S_BET_* is the Brunauer-Emmett-Teller (BET) surface area. ^b^ S*_ext_* is the external surface area, determined according to the t-plot method. ^c^ *V_total_* is the total pore volume of pores at a relative pressure (P/P_0_) of 0.99. ^d^ *V_micro_* is the micropore volume calculated from t-plot method. ^e^ *V_meso_* is the mesopore volume calculated by using $V_{total}-V_{micro}=V_{meso}$.

**Table 4:** The diffusion coefficient of toluene on the Pt/ZF-D, Pt/ZF, Pt/ZSM-5-D, and Pt/ZSM-5 catalysts at 30 °C.

Sample	P(mbar)	D_ads_ × 10^−19^ (m^2^/s)	R ^a^	SD ^b^
Pt/ZF-D	2	82.5	0.999	0.011
Pt/ZF	2	39.6	0.999	0.0092
Pt/ZSM-5-D	2	4.06	0.996	0.136
Pt/ZSM-5	2	3.45	0.997	0.267
Pt/ZF-D	1	69.5	0.999	0.003
Pt/ZF	1	26.8	0.999	0.008
Pt/ZSM-5-D	1	4.79	0.999	0.0098
Pt/ZSM-5	1	4.06	0.997	0.259

^a^ R: correlation coefficient. ^b^ SD: standard deviation of the fit.

## Supplementary Materials

The following are available online at https://www.mdpi.com/2079-4991/10/1/30/s1, Figure S1 (a) The CO_2_ yields during the toluene catalytic combustion. (b) The selectivity of CO_2_ in the toluene catalytic combustion products. Figure S2. The SEM characterization of the (a–b) fresh and (c–d) spent (after 50 h reaction time) 0.5%Pt/ZF-D catalysts. Figure S3 (1) (a) TEM images and (b) Pt particle size dispersion of (A) 0.1%Pt/ZF-D, (B) 0.5%Pt/ZF-D, (C) 1%Pt/ZF-D, and (D) 2%Pt/ZF-D; (2) XPS spectra of (a) 0.1%Pt/ZF-D, (b) 0.5%Pt/ZF-D, (c) 1%Pt/ZF-D, and (d) 2%Pt/ZF-D. Figure S4 (a) XRD patterns of the fresh and spent 0.5%Pt/ZF-D catalysts. (b) The pore width distribution curves of in the fresh and spent 0.5%Pt/ZF-D catalysts, respectively. Figure S5 (A) SEM images of ZSM-5, (B) SEM images of ZSM-5-D. Figure S6 (A) TEM images and Pt distribution of Pt/ZF-D, (B) TEM images and Pt distribution of Pt/ZF, (C) TEM images and Pt distribution of Pt/ZSM-5, (D) TEM images and Pt distribution of Pt/ZSM-5-D. Figure S7 pore width distribution curves of ZF, ZF-D, ZSM-5, ZSM-5-D. Table S1 ICP quantification results of the four catalysts. Table S2 Catalytic performance for toluene combustion over the Pt/ZF-D, Pt/ZF, Pt/ZSM-5-D, and Pt/ZSM-5 catalysts. Table S3 Kinetics for toluene reaction over the Pt/ZF-D, Pt/ZF, Pt/ZSM-5-D, and Pt/ZSM-5 catalysts. Table S4 Conversion of toluene combustion and textural parameters of various samples. Table S5 XPS results of the various catalysts.
