# Supplementary material for: Catalytic Performance of Toluene Combustion over Pt Nanoparticles Supported on Pore-Modified Macro-Meso-Microporous Zeolite Foam
Source: Nanomaterials (Basel). 2019 Dec 20;10(1):30. doi: 10.3390/nano10010030 (PMC7023492; doi:10.3390/nano10010030)
Supplement: Supplementary file 1 [file nanomaterials-10-00030-s001.pdf]

Supplementary Materials

Catalytic Performance of Toluene Combustion over Pt Nanoparticles Supported on Pore-Modified Macro-Meso-Microporous Zeolite Foam

Sibei Zou [1], Mingyuan Zhang [1], Shengpeng Mo [1], Hairong Cheng [1], Mingli Fu [1,2,3], Peirong Chen [1,2,3], Limin Chen [1,2,3], Wei Shi [1,*] and Daiqi Ye [1,2,3,*]

1 School of Environment and Energy, South China University of Technology, Guangzhou 510006, China; es2017zousibei@mail.scut.edu.cn (S.Z.); 201710106086@mail.scut.edu.cn (M.Z.); moshengpeng14@mails.ucas.ac.cn (S.M.); mschenghairong@mail.scut.edu.cn (H.C.); mlfu@scut.edu.cn (M.F.); chenpr@scut.edu.cn (P.C.); liminchen@scut.edu.cn (L.C.)

2 National Engineering Laboratory for VOCs Pollution Control Technology and Equipment, Guangzhou Higher Education Mega Centre, Guangzhou 510006, China

3 Guangdong Provincial Key Laboratory of Atmospheric Environment and Pollution Control (SCUT), Guangzhou Higher Education Mega Centre, Guangzhou 510006, China

* Correspondence: sww@scut.edu.cn (W.S.); cedqye@scut.edu.cn (D.Y.)

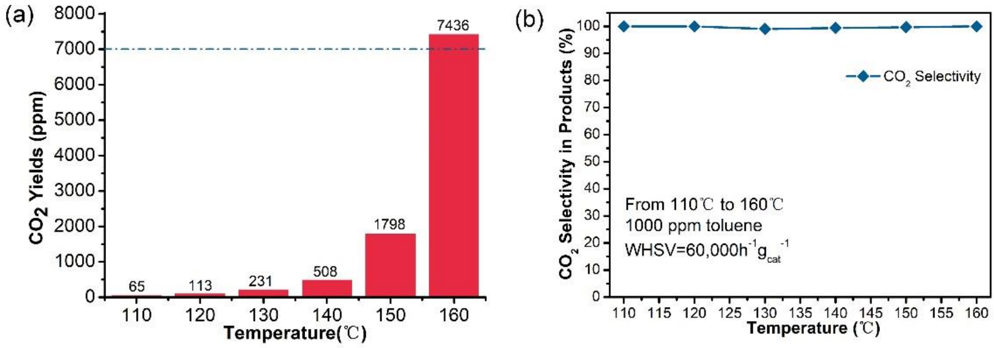

Figure S1. (a) The CO_2 yields during the toluene catalytic combustion. (b) The selectivity of CO_2 in the toluene catalytic combustion products.

According to the carbon balance during the toluene catalytic combustion, if 1000 ppm toluene was completely conversed into CO_2, the yields of CO_2 might be 7000 ppm in ideal situation. And during the reaction period, the concentration of CO_2 production would always be 7 times the concentration of toluene degradation. In this case, it is shown in Figure. S1 that the concentration of CO_2 was 7436 ppm at 160 °C, which was approximately 7 times of the concentration of toluene. And the CO_2 selectivity in Figure. S2 during toluene combustion was 99-100%. It is indicated that the only products of toluene combustion in our case were CO_2 and H_2O and no CO was detected during the combustion.

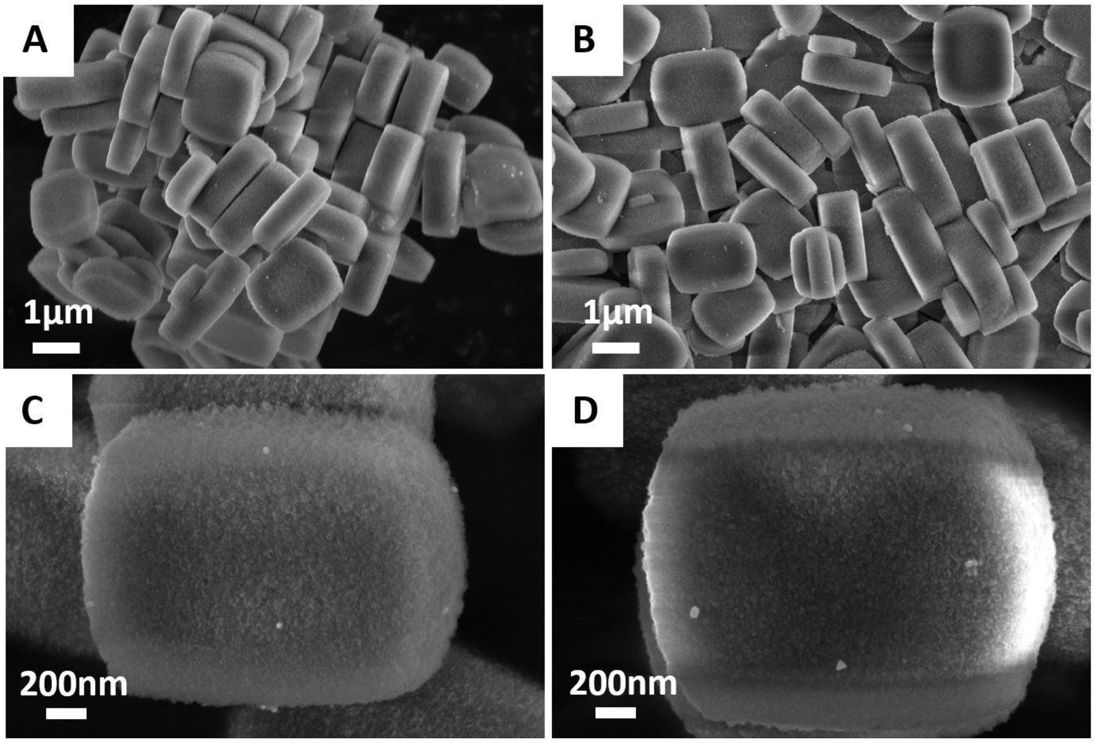

Figure S2. The SEM characterization of the (a) (b) fresh and (c) (d) spent (after 50 h reaction time) 0.5%Pt/ZF-D catalysts.

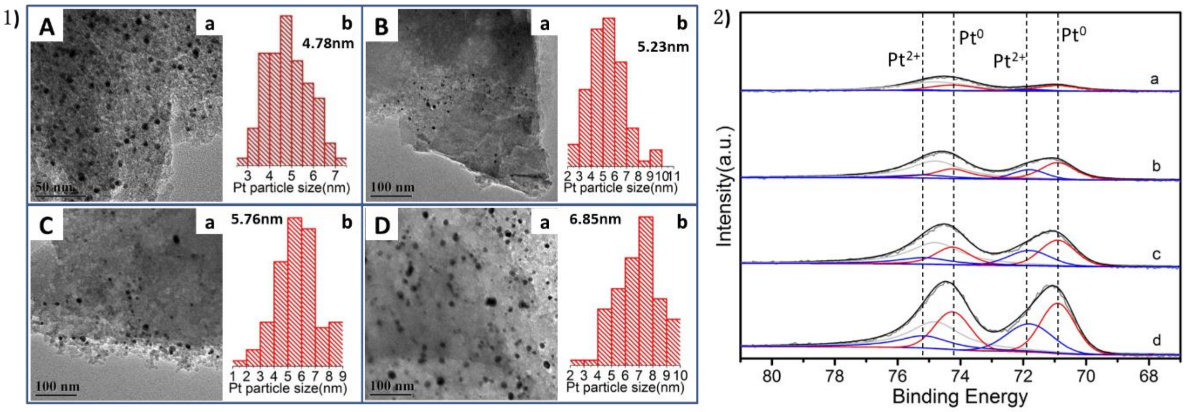

Figure S3. 1) (a) TEM images and (b) Pt particle size dispersion of (A) 0.1%Pt/ZF-D, (B) 0.5%Pt/ZF-D, (C) 1%Pt/ZF-D, and (D) 2%Pt/ZF-D; 2) XPS spectra of (a) 0.1%Pt/ZF-D, (b) 0.5%Pt/ZF-D, (c) 1%Pt/ZF-D, and (d) 2%Pt/ZF-D.

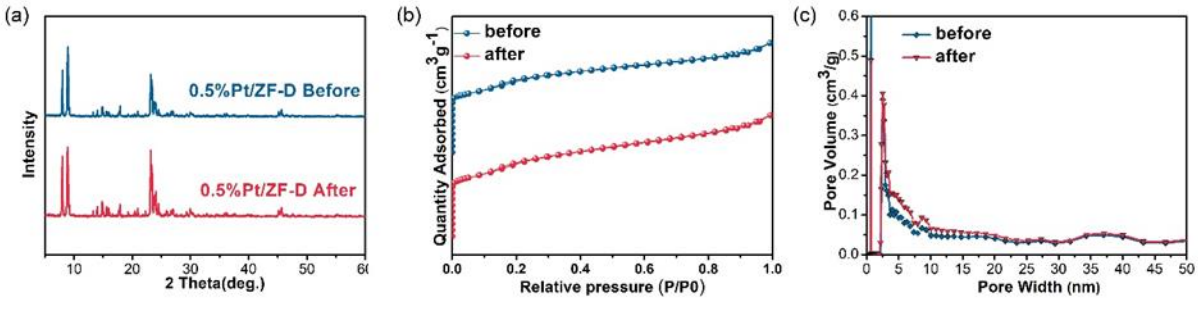

Figure S4. (a) XRD patterns of the fresh and spent 0.5%Pt/ZF-D catalysts. (b) The pore width distribution curves of in the fresh and spent 0.5%Pt/ZF-D catalysts, respectively.

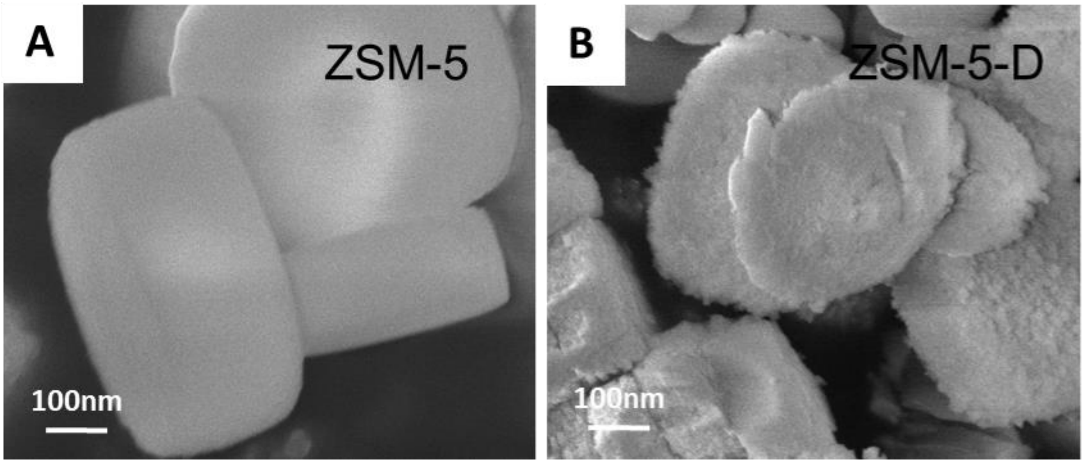

Figure S5. (A) SEM images of ZSM-5, (B) SEM images of ZSM-5-D.

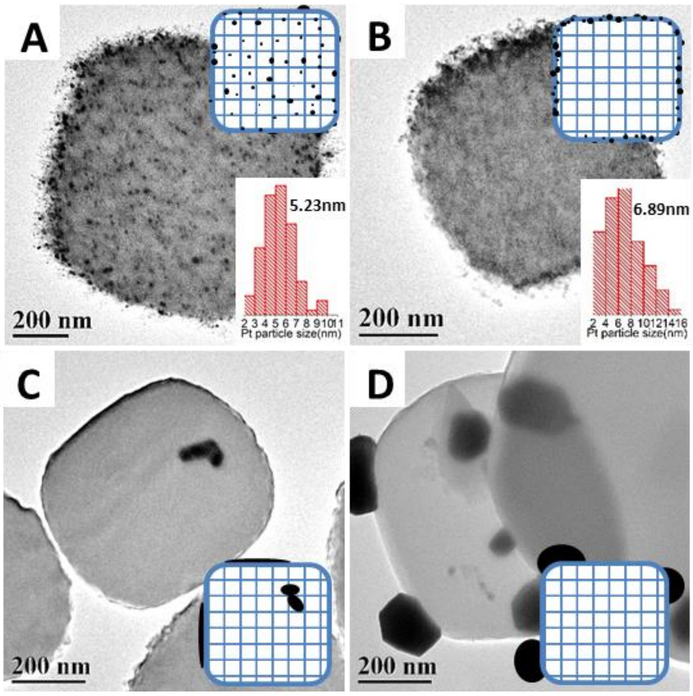

Figure S6. (A) TEM images and Pt distribution of Pt/ZF-D, (B) TEM images and Pt distribution of Pt/ZF, (C) TEM images and Pt distribution of Pt/ZSM-5, (D) TEM images and Pt distribution of Pt/ZSM-5-D.

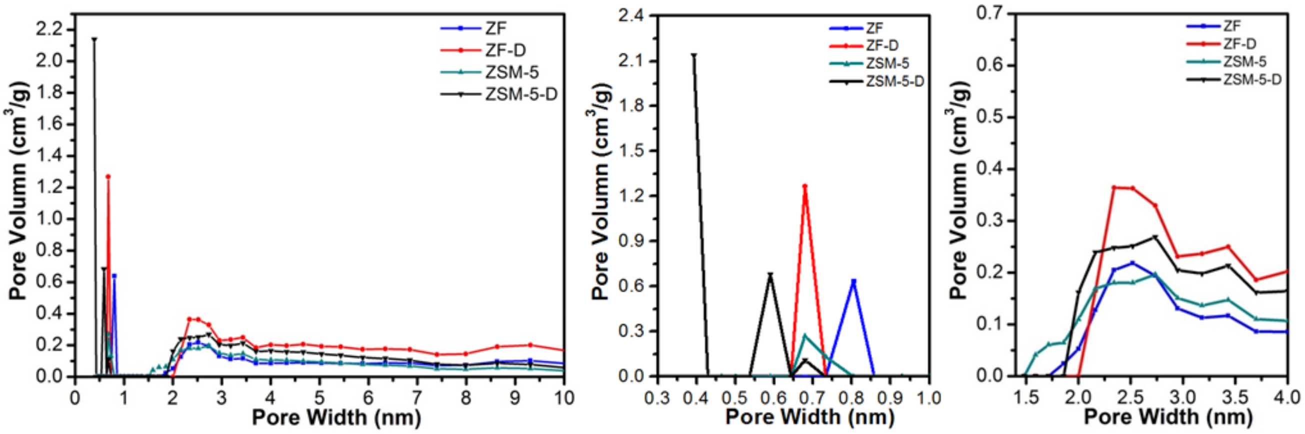

Figure S7. pore width distribution curves of ZF, ZF-D, ZSM-5, ZSM-5-D.

Table S1. ICP quantification results of the four catalysts.

Catalysts	Pt(wt.%)	Si/Al Molar ratio	Increased ratio of Si/Al
Pt/ZF-D	0.52	84	58.59%
Pt/ZF	0.53	53	
Pt/ZSM-5-D	0.51	74	25.42%
Pt/ZSM-5	0.54	59	

Table S2. Catalytic performance for toluene combustion over the Pt/ZF-D, Pt/ZF, Pt/ZSM-5-D, and Pt/ZSM-5 catalysts.

Catalysts	Activities (℃)			E_a (kJ/mol)[a]	R^2 [b]	$rate_{Toluene}$ (10^{-7}mol /s·g)[c]	TOF ($10^{-3}S^{-1}$)[d]
	T_5	T_{50}	T_{90}				
Pt/ZF-D	135	152	158	84	0.997	4.64	4.21
Pt/ZF	156	172	178	99	0.997	1.16	1.38
Pt/ZSM-5-D	158	173	179	106	0.998	1.67	7.25
Pt/ZSM-5	164	183	188	108	0.995	0.91	8.08

[a] E_a values were calculated under 10% conversion rates of toluene.

[b] R^2 values were the correlation coefficients of Arrhenius plots.

[c] $rate_{Toluene}$ values were calculated from the Eq. (4) at the 140℃ under 10% conversion of toluene.

[d] TOF values were calculated from the Eq. (5) at 140℃.

Table S3. Kinetics for toluene reaction over the Pt/ZF-D, Pt/ZF, Pt/ZSM-5-D, and Pt/ZSM-5 catalysts.

Catalysts	1000/T (K^{-1})	$r_{Toluene}$[a] (10^{-7}mol/s·g)	lnr[b] (mol/s·g)	Arrhenius Plot[c]	E_a (kJ/mol)
Pt/ZF-D	2.61	0.68	−16.5	y = −10.12x + 9.9045	84
	2.54	1.24	−15.9		
	2.48	2.37	−15.3		
	2.42	4.64	−14.6		
Pt/ZF	2.54	0.28	−17.4	y = −11.97x + 13.086	99
	2.48	6.32	−16.6		
	2.42	1.16	−15.9		
	2.36	2.54	−15.2		
Pt/ZSM-5-D	2.48	0.76	−16.4	Y = −12.75x+15.251	106
	2.42	1.67	−15.6		
	2.36	3.45	−14.9		
	2.31	6.84	−14.2		
Pt/ZSM-5	2.42	0.91	−16.2	Y = −12.97+15.225	108
	2.36	1.99	−15.4		
	2.31	4.32	−14.6		
	2.26	7.47	−14.1		

[a] $rate_{Toluene}$ values were calculated from the Eq. (4) all under 10% conversion of toluene.

[b] lnr values were calculated from the equation mentioned below.

[c] Arrhenius Plots were the liner fitting plot of 1000/T and lnr.

It has been recognized that catalytic combustion of toluene in the presence of excess oxygen is first-order and zero-order kinetics with respect to toluene concentration (c) and oxygen concentration, respectively[1]. Therefore, the equation is as follows:

$$\mathrm{r} = -\mathrm{kc} = \left[-A \ exp\left(\frac{-Ea}{RT}\right)\right] c$$

where r, k, A, and E_a stand for the reaction rate [mol/(g s)], rate constant (s^{-1}), pre-

exponential factor (s^{-1}), and apparent activation energy (kJ/mol), respectively. The k values could be calculated by the reaction rates and reactant conversions.

Table S4. Conversion of toluene combustion and textural parameters of various samples.

Catalysts	T_{50} /℃	T_{100} /℃	Pt loadings[a]/%	Pt particle size[b]/nm	Pt dispersion[b]/%	$P(Pt^0)$[c]/%
0.1%Pt/ZF-D	194	205	0.084	4.78	23.5	83
0.5%Pt/ZF-D	152	158	0.58	5.23	21.5	67
1%Pt/ZF-D	144	148	1.02	5.76	19.5	59
2%Pt/ZF-D	141	148	2.05	6.85	16.4	58

a Determined by ICP-OES.
b Determined by TEM.
c Determined by XPS.

Table S5. XPS results of the various catalysts[a].

Catalysts	Pt species	Peak area $Pt4f_{7/2}$	Peak area $Pt4f_{5/2}$	$P(Pt^0)$[b]
Pt/ZF-D	Pt^0	2207	1655	67.3%
	Pt^{2+}	1072	804	
Pt/ZF	Pt^0	2522	1892	65.1%
	Pt^{2+}	1348	1011	
Pt/ZSM-5-D	Pt^0	3438	2578	62.2%
	Pt^{2+}	2407	1805	
Pt/ZSM-5	Pt^0	567	425	46.6%
	Pt^{2+}	344	258	

[a] Determined from XPS measurements (Figure.9).
[b] Proportion of Pt^0=Area(Pt^0)/[Area(Pt^0)+ Area(Pt^{2+})]×100%.

For the activity of the catalyst is only slightly improved with the Pt loading from 1% to 2%, the mean size of Pt particle of 0.1%, 1%, and 2% Pt/ZF-D is 4.76nm, 5.76nm, and 6.85nm with Pt dispersion of 23.5%, 19.5%, and 16.4% respectively (Figure. S1). The larger the Pt particles, the lower the Pt dispersion of the catalyst has. This might be consistent with the fact that as the Pt loading increases, the increase in catalyst activity is less obvious (Figure. 2).

The mathematical solution for the transient diffusion equation for a spherical particle assumes as[2]:

$$\frac{Q_t - Q_0}{Q_\infty - Q_0} = 1 - \frac{6}{\pi^2}\sum_{n=1}^{\infty}\left[\frac{1}{n^2}\left(-\frac{n^2\pi^2 t}{r_0^2}D\right)\right] \tag{1}$$

Where Q_t, Q_∞ and Q_0 are the amounts adsorbed at time t, at time of sorption equilibrium and at time t = 0, respectively. For short time periods, Eq. (1) approaches the expression:

$$\frac{Q_t - Q_0}{Q_\infty - Q_0} = \frac{6}{r_0}\sqrt{\frac{Dt}{\pi}} \tag{2}$$

Where D is the diffusion coefficient of toluene on the prepared catalysts, r_0 is the radius of catalysts, in our research, r_0 of Pt/ZF-D and Pt/ZF is 750nm, and r_0 of Pt/ZSM-5-D and Pt/ZSM-5 is 200 nm.

References

[1] C.T. Wong, A.Z. Abdullah, S. Bhatia, Catalytic oxidation of butyl acetate over silver-loaded zeolites, Journal of Hazardous Materials, 157 (2008) 480-489.
[2] L. Zhao, B. Shen, J. Gao, C. Xu, Investigation on the mechanism of diffusion in mesopore structured ZSM-5 and improved heavy oil conversion, Journal of Catalysis, 258 (2008) 228-234.
